# The metabolomics approach revealed a distinctive metabolomics pattern associated with hyperthyroidism treatment

**DOI:** 10.3389/fendo.2022.1050201

**Published:** 2022-11-10

**Authors:** Malak A. Jaber, Hicham Benabdelkamel, Lina A. Dahabiyeh, Afshan Masood, Reem H. AlMalki, Mohthash Musambil, Assim A. Alfadda, Anas M. Abdel Rahman

**Affiliations:** ^1^ Pharmaceutical Medicinal Chemistry and Pharmacognosy, Faculty of Pharmacy and Medical Sciences, University of Petra, Amman, Jordan; ^2^ Proteomics Resource Unit, Obesity Research Center, College of Medicine, King Saud University, Riyadh, Saudi Arabia; ^3^ Division of Pharmaceutical Sciences, School of Pharmacy, The University of Jordan, Amman, Jordan; ^4^ Metabolomics Section, Department of Clinical Genomics, Center for Genome Medicine, King Faisal Specialist Hospital and Research Centre (KFSHRC), Riyadh, Saudi Arabia; ^5^ Department of Botany and Microbiology, College of Science, King Saud University, Riyadh, Saudi Arabia; ^6^ Department of Medicine, College of Medicine and King Saud Medical City, King Saud University, Riyadh, Saudi Arabia; ^7^ Department of Biochemistry and Molecular Medicine, College of Medicine, Alfaisal University, Riyadh, Saudi Arabia; ^8^ Department of Chemistry, Memorial University of Newfoundland, St. John’s, NL, Canada

**Keywords:** hyperthyroidism, carbimazole, metabolomics, acylcarnitines, tryptophan metabolism, redox hemostasis, biomarkers

## Abstract

**Background:**

Hyperthyroidism is characterized by increased thyroid hormone production, which impacts various processes, including metabolism and energy expenditure. Yet, the underlying mechanism and subsequent influence of these changes are unknown. Metabolomics is a broad analytical method that enables qualitative and quantitative examination of metabolite level changes in biological systems in response to various stimuli, pathologies, or treatments.

**Objectives:**

This study uses untargeted metabolomics to explore the potential pathways and metabolic patterns associated with hyperthyroidism treatment.

**Methods:**

The study consisted of 20 patients newly diagnosed with hyperthyroidism who were assessed at baseline and followed up after starting antithyroid treatment. Two blood samples were taken from each patient, pre (hyperthyroid state) and post-treatment (euthyroid state). Hyperthyroid and euthyroid states were identified based on thyroxine and thyroid-stimulating hormone levels. The metabolic alteration associated with antithyroid therapy was investigated using liquid chromatography- high-resolution mass spectrometry. The untargeted metabolomics data was analyzed using both univariate and multivariate analyses using MetaboAnalyst v5.0. The significant metabolic pattern was identified using the lab standard pipeline, which included molecular annotation in the Human Metabolome Database, LipidMap, LipidBlast, and METLIN. The identified metabolites were examined using pathway and network analyses and linked to cellular metabolism.

**Results:**

The results revealed a strong group separation between the pre- and post-hyperthyroidism treatment (Q2 = 0.573, R2 = 0.995), indicating significant differences in the plasma metabolome after treatment. Eighty-three mass ions were significantly dysregulated, of which 53 and 30 characteristics were up and down-regulated in the post-treatment compared to the pre-treatment group, respectively. The medium-chain acylcarnitines, octanoylcarnitine, and decanoylcarnitine, previously found to rise in hyperthyroid patients, were among the down-regulated metabolites, suggesting that their reduction could be a possible biomarker for monitoring euthyroid restoration. Kynurenine is a downregulated tryptophan metabolite, indicating that the enzyme kynurenine 3-hydroxylase, inhibited in hyperthyroidism, is back functioning. L-cystine, a cysteine dimer produced from cysteine oxidation, was among the down-regulated metabolites, and its accumulation is considered a sign of oxidative stress, which was reported to accompany hyperthyroidism; L-cystine levels dropped, this suggests that the plasma level of L-cystine can be used to monitor the progress of euthyroid state restoration.

**Conclusion:**

The plasma metabolome of patients with hyperthyroidism before and after treatments revealed differences in the abundance of several small metabolites. Our findings add to our understanding of hyperthyroidism’s altered metabolome and associated metabolic processes and shed light on acylcarnitines as a new biomarker for treatment monitoring in conjunction with thyroxine and thyroid-stimulating hormone.

## Graphical Abstract

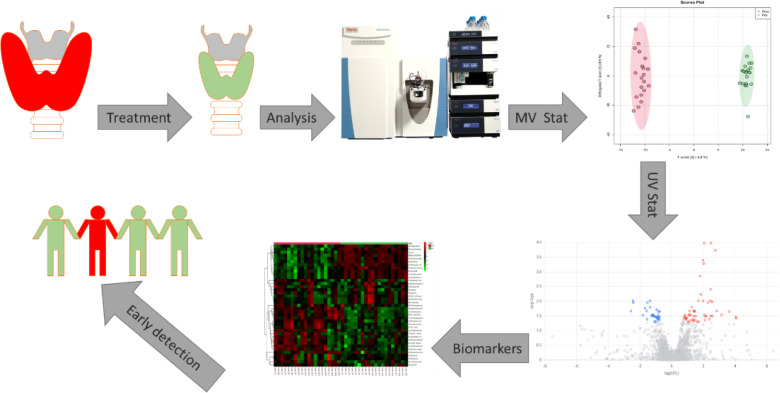

## Introduction

The thyroid gland is a small, butterfly-shaped endocrine gland near the front of the neck. Thyroxine (FT4) is considered the main hormone secreted by the thyroid gland, and it is activated mainly in the liver and the brain into triiodothyronine (FT3) by the removal of an iodine atom (1). Thyroid-stimulating hormone (TSH), released from the pituitary gland, regulates the amount of FT4 released, controlled by thyrotrophin-releasing hormone (TRH) released from the hypothalamus to maintain the correct balance of these hormones. FT4 and FT3 play a key role in maintaining the body’s main functions by regulating body temperature, cardiovascular function, bone health, and metabolism. Therefore, any misbalance in their levels will lead to serious dysfunction in the body (2).

Hyperthyroidism (HT) (or overactive thyroid) is an endocrine disorder characterized by excessive synthesis and secretion of thyroid hormones. Hyperactivity, heat intolerance, weight loss, tremors, anxiety, nervousness, increased perspiration, palpitations, and hair loss are common clinical signs of HT. Still, the clinical picture may be less obvious in senior patients (3). Biochemically, HT is confirmed by abnormal levels of FT3 and FT4 and below-normal levels of TSH (4). HT crucially affects various organs and pathways in the body and greatly impacts growth and development, metabolism, energy storage, and expenditure (5, 6). In addition to HT’s direct effect on lipids, the level of carbohydrates and amino acids metabolism increases both gluconeogenesis and glycolysis (7). Therefore, early HT diagnosis is essential to enable healthcare professionals to provide the proper treatment at an early stage (8).

Metabolomics is increasingly applied as a new advanced analytical approach for discovering prognostic and diagnostic biomarkers of various diseases (9) and monitoring the therapeutic effects (10, 11). Metabolomics, also known as metabolome analysis, uses various experimental, statistical, and computational approaches to study perturbation in small metabolites (MW <1500 Da), including sugars, amino acids, nucleotides, oligonucleotides, lipids, steroids, and peptides and their alterations in a living organism under different circumstances. Mass spectrometry (MS) and nuclear magnetic resonance spectroscopy (NMR) are metabolomics’ second most commonly used techniques hyphenated to a chromatographic system is preferable due to their inherent high sensitivity detection, quantitation, and structure elucidation of hundreds of metabolites in a single run (12).

In HT, metabolomics can provide a deeper understanding of HT pathophysiology and how the disease affects small intra and extracellular metabolites. This approach could improve our understanding of disease phenotypes, aid in discovering new biomarkers for disease diagnosis and progression, identify novel therapeutic targets, and enable appropriate treatments and proper monitoring (13). The metabolic disturbances associated with HT are poorly understood. This study used LC-MS to identify biomarkers that could distinguish between euthyroid and hyperthyroid states, allowing for accurate individual thyroid function prediction independent of TSH and FT4 measurement, and molecular characterization of pre-clinical hyperthyroidism. Limited literature reported the metabolomics changes associated with HT and the effect of antithyroid treatment. Previous studies used a metabolomics approach to investigate the metabolomic changes taking place upon the transition from HT to euthyroidism with medical treatment (14) and examine changes in metabolism associated with reinstatement of euthyroidism in Swedish patients (15). However, the metabolomic profile of treated HT patients is profoundly altered by hyperthyroidism, according to Piras and his colleagues, and this disruption endures even after the euthyroid condition is reached. This finding shows that standard tests like TSH, FT3, and FT4 assays may not be insufficient to identify the long-term consequences of thyroid hormones (16).

To shed further light on alterations in metabolism related to the re-establishment of euthyroidism in the population of Western Asia compared to the Chinese and Swedish populations, metabolite profiling of plasma extract using LC-MS of HT patients from Saudi Arabia before and after treatment of HT was explored and discussed.

## Material and methods

### Study design

Twenty newly diagnosed patients with HT referred to the King Khaled University Hospital’s (KKUH) endocrine outpatient clinic, were enrolled in this study. Low levels of TSH (below 0.25 mIU/L) and a high level of FT4 in the blood (greater than 22 pmol/L) were used to define HT. The Institutional Review Board (IRB) of King Saud University’s: College of Medicine in Riyadh, Saudi Arabia (registration No E-10-172) reviewed and approved the study. All patients signed informed consent. Among the 20 recruited patients 8 males and 12 females were recruited and their average age was 40 ± 11.5 years. All patients were healthy and did not have any other significantly associated pathological conditions such as type 2 diabetes mellitus, hypertension, inflammatory or autoimmune conditions. Patients were treated with an appropriate dose of the antithyroid medication, carbimazole (CBZ) 20 mg daily which was gradually tapered according to the FT4 levels. The patients were monitored after CBZ therapy until an erythroid state was achieved, which is defined as a normal level of FT4 and TSH.

Blood samples were collected pre and post-treatment from each patient. Blood samples were collected after overnight fasting using EDTA-coated tubes (BD Vacutainer, USA). Plasma was obtained by centrifugation for 15 min at 3000xg, and stored at -80°C for further analysis.

### Biochemical analysis

A Dimension^®^ Xpand Plus integrated clinical chemistry autoanalyzer (Siemens Healthcare Diagnostics, Deerfield, IL, USA) was used to perform and determine all parameters for biochemical and hormone studies listed in **Table 1**. The Friedewald equation was used to calculate the serum levels of low-density lipoprotein (LDL) cholesterol (17).

**Table 1:** Biochemical profile of the hyperthyroid patients’ pre- and post-treatment using carbimazole as antithyroid medication.

Parameters	Hyperthyroid	Euthyroid	*p-*value
Patients number	20		
Age (years)	40 ± 11.5		
Glucose (mmol/L)	5.5 ± 0.7	5.6 ± 0.6	0.63
Urea (mmol/L)	4.6 ± 0.5	4.8 ± 0.5	0.21
Creatinine (umol/L)	62.1 ± 10.7	65.8 ± 11.0	0.45
Sodium (mmol/L)	139.2 ± 1.9	139.1 ± 0.4	0.81
Potassium (mmol/L)	4.4 ± 0.1	4.4 ± 0.2	1.00
Aspartate transaminase (IU/L)	33.9 ± 8.9	30.6 ± 4.2	0.30
Alanine transaminase (IU/L)	17.1 ± 4.8	15.2 ± 1.9	0.25
Alkaline phosphatase (IU/L)	113.9 ± 52.9	119.8 ± 28.4	0.76
FT4 (pmol/L)	34.9 ± 9.8	16.9 ± 2.9	< 0.0001^*^
TSH (mIU/L)	0.014 ± 0.02	0.7 ± 0.5	< 0.0001^*^
Total cholesterol (mmol/L)	4.5 ± 1.2	4.8 ± 1.0	0.55
LDL cholesterol (mmol/L)	2.6 ± 0.8	3.0 ± 0.8	0.27
TG (mmol/L)	1.0 ± 0.7	0.8 ± 0.5	0.31
HDL cholesterol (mmol/L)	1 ± 0.3	1.3 ± 0.3	0.03^*^

^*^Indicate statistically significant alterations (p-value < 0.05).

FT4, free thyroxine; TSH, thyroid-stimulating hormone; LDL, low-density lipoprotein; TG, triglycerides; HDL, high-density lipoprotein.

### Metabolites extraction and LC-MS analysis

Plasma metabolites were extracted using a standard procedure, including protein precipitation using a mixture of methanol and acetonitrile (1:1), vortexing in Thermomixer (Eppendorf, Germany) at 600 rpm, 4°C for 1 h., then centrifugation at 12000 x g for 10 min at 4°C. After that, the supernatant was dried under vacuum and resuspended using 50% mobile phase A: B (A: 0.1% Formic acid in H_2_O), (B: 0.1% Formic acid in 50% MeOH and ACN). QC samples were prepared with aliquots from all samples to check for system stability. Metabolic fingerprints were explored using the Waters Acquity UPLC system coupled with a Xevo G2-S QTOF mass spectrometer equipped with an electrospray ionization source (ESI). The extracted metabolites were chromatographed using an ACQUITY UPLC using XSelect (100×2.1mm 2.5 μm) column (Waters Ltd., Elstree, UK), the mobile phase composed of 0.1% formic acid in H_2_O as solvent A, and B consists of 0.1% formic acid in 50% ACN: MeOH. A gradient elution schedule was run as follows: 0-16 min 95- 5% A, 16-19 min 5% A, 19-20 min 5-95% A, 20-22 min 95- 95% A, at 300 µL/min flow rate. MS spectra were acquired under positive and negative electrospray ionization modes (ESI+, ESI-). MS conditions were as follows: source temperature was 150°C, the desolvation temperature was 500°C (ESI+) or 140 (ESI−), the capillary voltage was 3.20 kV (ESI+) or 3 kV (ESI−), cone voltage was 40 V, desolvation gas flow was 800.0 L/h, cone gas flow was 50 L/h. The collision energies of low and high function were set off at 10 V and 50 V respectively, in MSE mode. The mass spectrometer was calibrated with sodium formate in 100–1200 Da. Data were collected with Masslynx™ V4.1 workstation in continuum mode.

### Data processing and statistical analysis

Peak picking and alignment of detected ion (m/z, Rt) were processed using Progenesis QI software from Waters (Waters Technologies, Milford, MA., USA). Then, the processed data were statistically evaluated using MetaboAnalyst v5.0 (McGill University, Montreal, QC, Canada) (18). Firstly, data were subjected to log-transformation, mean centering, and Pareto scaling, followed by univariate and multivariate analyses. In multivariate analysis, orthogonal partial least square discriminative analysis (OPLS-DA) was used to extract significantly perturbed metabolites between the pre and post-treatment groups. Features with variable importance in the projection (VIP) value >1 were considered significant. In univariate analysis, paired t-test was used to compare the two groups and metabolites with a false discovery rate (FDR) < 0.05 were considered significant. Significantly altered features were chosen based on univariate and multivariate analyses. Pathway analysis uncovered important metabolic changes, features, or biomarkers linked with antithyroid therapy. In addition, Receiver Operating Characteristic (ROC) curves were created using the PLS-DA approach in the MetaboAnalyst v 5.0 for global analysis to identify possible biomarkers. Metabolites were putatively identified based on the exact mass searched against different databases including Human Metabolome Database, LipidMap, LipidBlast, and METLIN.

## Results

### Biochemical analysis

**Table 1** summarizes the study participants’ characteristics and biochemical tests. After antithyroid medication, the FT4 level was significantly decreased while the level of TSH and serum high-density lipoprotein (HDL) was significantly increased (p-value < 0.05), which is consistent with previous report (18). Other biochemical profiles, including glucose, liver enzymes, and lipid profile, were not statistically significant after antithyroid treatment, **Table 1**.

### Mass ion detection and metabolites identification

A total of 12522 mass ion features were detected in both positive and negative ionization modes. After several filtration processes such as alignment, peak picking, and missing value removal, 2499 features were retained for statistical analysis. To confirm that all depicted data has a Gaussian distribution, the data were normalized by the median, log-transformed and Pareto scaled to eliminate systemic variances.

**Figure 1:**
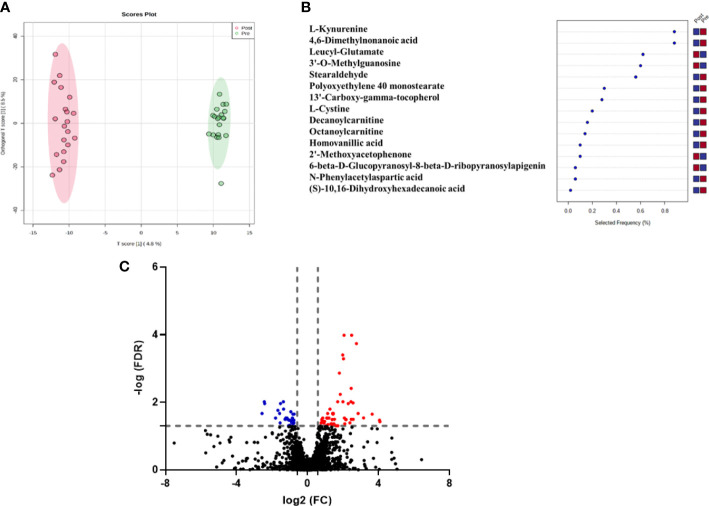
**(A)** OPLS-DA model for the 2499 feature showed evident separation between pre and post-antithyroid drug administration groups. The robustness of the created models was evaluated by the fitness of the model (R2Y=0.995) and predictive ability (Q2 = 0.573) values, **(B)** The loading plot depicts the modulation of metabolic expression of metabolites between pre and post-antithyroid drug administration groups (red indicates higher and blue indicates lower), **(C)** Volcano plot shows the up (n=53) and down (n=30) regulated metabolites in post-treatment compared to the initial samples (The fold change (FC**)** and FDR (adjusted p-value) cutoffs were 1. 5 and 0.05, respectively).

**Figure 1A** shows the variability between the pre and post-treatment groups using OPLS-DA. The apparent separation in the score plot reflects the difference between the two groups, with a computed R2 = 0.995 and Q2 = 0.573. A total of 2499 feature ions were responsible for the difference in metabolomics expression levels across groups and thus were responsible for the group separation in the OPLS-DA. The selected frequency percent score was calculated using OPLS-DA data to determine the contribution levels of various metabolites. This could pave the way for the discovery of biomarkers associated with HT. L-Kynurenine, L-Cystine, Decanoylcarnitine, and Octanoylcarnitineare shown in the selected frequency plot (**Figure 1B**).

**Figure 2:**
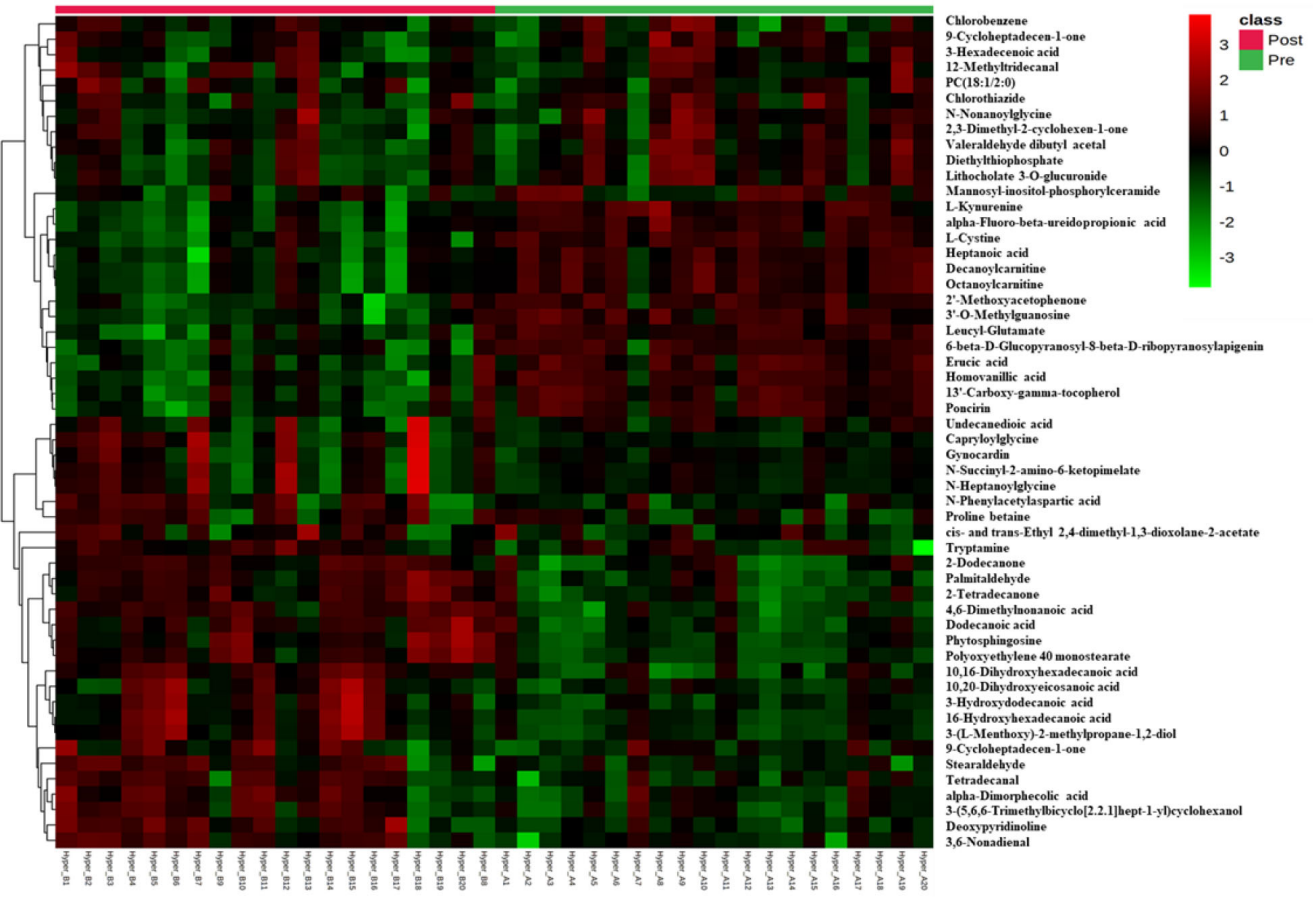
Heatmap of untargeted LC-MS analysis of biologically relevant and significantly altered metabolites between pre and post-antithyroid drug administration groups. Red and green colors refer to significantly up- and down-regulated metabolites, respectively.

Univariate analysis was used to identify significantly altered features between the two groups. Volcano Plot using FDR p-value ≤ 0.05 and fold change cutoff of 1.5 revealed that eighty-three mass ions were significantly dysregulated (**Figure 1C**). In the post-treatment group compared to the pre-treatment group, 53 and 30 features of the eight-three mass ions, respectively, were up- and down-regulated. Among these features, 53 metabolites were identified and are shown in **Table S1**, of which 38 and 15 were up and down-regulated, respectively. The heat map (**Figure 2**) depicts the relative concentrations of metabolites in pre and post-treatment groups, with red and green color intensities indicating the relative increase and decrease in metabolite concentrations, respectively.

**Figure 3:**
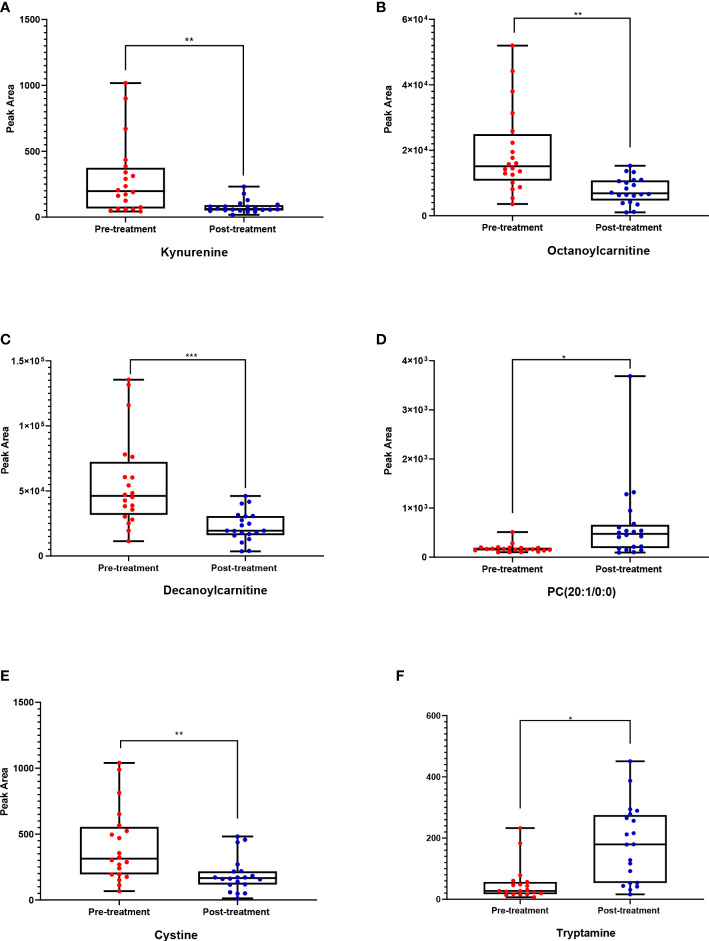
Box whisker plots for six putatively identified compounds for pre-treatment (red) (n = 20 volunteers) and post-treatment (blue) cohorts (n = 20). Where **(A)** Kynurenine, **(B)** Octanoylcarnitine, **(C)** Decanoylcarnitine, **(D)** PC (20:1/0:0), **(E)** Cystine, **(F)** Tryptamine. The significance represents the difference between pre- and post-treatment samples, where * = significant (p < 0.05), ** = very significant (p < 0.01), and *** = highly significant (P ≤ 0.001).

These metabolites might be considered promising biomarkers for transitioning from HT to euthyroid state following treatment with carbimazole. Among these metabolites, kynurenine, cystine, octanoylcarnitine, and decanoylcarnitine were downregulated while PC (20:1) and tryptamine were upregulated in the post-treatment group, as displayed in **Figure 3**. These disturbed metabolites were subjected to pathway analysis to identify the most altered pathways. Fatty acid biosynthesis, tryptophan metabolism, and biosynthesis of unsaturated fatty acids were among the most affected pathways between the two groups.

**Figure 4:**
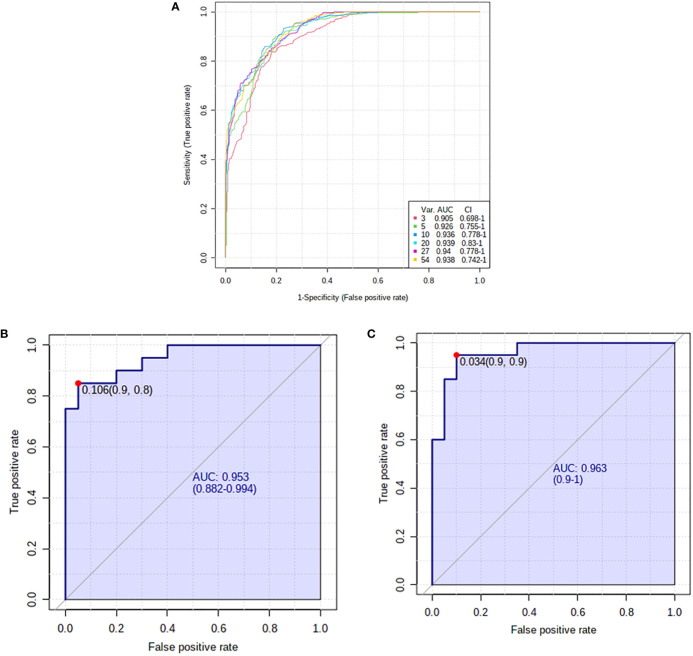
Receiver operating characteristics (ROC) curve and loading for significantly altered metabolites in pre- and post-hyperthyroid drug administration. **(A)** The PLS-DA model produced a ROC with an area under the curve (AUC) of at least 0.905 for the top 6 variants. **(B)** L-kynurenine (AUC-0.953) and **(C)** 3-O-Methylguanosine (AUC-0.963) were downregulated in post-treatment group. The median, log-transformed, and Pareto normalized data scaled to confirm that all depicted data has a Gaussian distribution.

The potential disease biomarkers were evaluated using Receiver Operating Characteristic (ROC) curve. PLS-DA was used as a classification and feature ranking approach to creating a multivariate exploratory ROC analysis. Six features at the exploratory ROC curve using PLS-DA with cross-validation (CV) had an Area Under the Curve (AUC) value of at least 0.909 (95% CI) (**Figure 4A**). For instance, the ROC curve’s AUC values for L-kynurenine (**Figure 4B**) and the modified purine nucleoside 3-O-methylguanosine (**Figure 4C**) were 0.953 and 0.963, respectively.

## Discussion

Hyperthyroidism has been associated with insulin resistance and weight loss, among other metabolic disorders. As a result, alterations in metabolism should accompany the re-establishment of euthyroidism. Because the metabolic consequences of HT are poorly understood, an unbiased metabolite profile was performed pre- and post-treatment to better understand the changes in patients’ plasma metabolome at the HT state. Patients may act as their control group because they were compared pre- and post-treatment. Another goal of this study was to identify hyperthyroidism-specific metabolic pathways that could be used in disease detection and treatment monitoring. Previous reports found that after three months of treatment, most metabolites that were affected showed altered levels and did not change further over the next 12 months. Therefore, in the present work, we focused on short-term changes that can be used to identify treatment monitoring biomarkers, and all samples were taken after the re-establishment of euthyroidism.

The adopted coupled univariate/multivariate statistics platforms showed that 53 metabolites were significantly altered after treatment initiation. These metabolites were mainly involved in fatty acid biosynthesis and metabolism. Octanoylcarnitine and decanoylcarnitine, intermediate chain acylcarnitines (C8–C14), were among the lower metabolites in the pre compared to post-treatment groups. Studies performed in both animal models and humans have linked acylcarnitine levels with FT4 levels in the serum. Serum FT4 levels under physiological conditions such as fasting and exercise were found to be associated with acylcarnitine levels (19). In addition, in a euthyroid population, levels of various acylcarnitines have been linked to FT4 (20). Thyroid hormone entry to the nucleus is known to be hampered by carnitine, resulting in decreased thyroid hormone function (21). Therefore, carnitine has been utilized in a few cases to alleviate clinical symptoms of thyroid storm and iatrogenic HT (22). It was reported that following treatment for HT, levels of intermediate-chain acylcarnitines are reduced (15). This class of acylcarnitines is derived from fatty acid metabolism and has been demonstrated to decline rapidly upon feeding after fasting (23). In the postprandial state, elevated plasma acylcarnitines correlate with plasma glucose. As a result, altered acylcarnitine levels are likely caused by increased glucose and lipid oxidation (24) and reduced glucose tolerance (25), both of which are linked to HT. As carnitine transports fatty acids in the form of acylcarnitine to the mitochondria, where fatty acid beta-oxidation takes place. Previous studies have shown that thyroid hormones boost fatty acid translocation to mitochondria and increase β-oxidation rate and oxygen consumption (26, 27). In HT, an increase in acylcarnitines level suggest an incomplete fatty acid oxidation due to an increase in beta-oxidation that exceed the capacity of tricarboxylic acid (TCA) cycle. As a result, acylcarnitines and reactive oxygen species build-up, which may result in proinflammatory pathways activation, impair insulin signaling, increased lipogenesis, metabolic stress, issue remodeling and organ dysfunction (28–30). However, upon treatment, the level of acylcarnitines were decreased which indicate complete fatty acid oxidation. This effect can be exploited to monitor HT treatment progress.

Tryptophan metabolism was affected by HT therapy. Tryptophan is an amino acid linked to aging rates metabolites that modulate inflammation, energy homeostasis, and behavior (31). Kynurenine, a key component of the tryptophan metabolic route, can readily cross the blood-brain barrier, where it either gets transformed into the neuroprotective kynurenic acid or the neurotoxic quinolinic acid (32). Disturbances in the equilibrium of these endogenous chemicals can be associated with many illnesses including stroke, epilepsy, multiple sclerosis, and amyotrophic lateral sclerosis, and neurological disorders including Alzheimer’s disease, Huntington’s disease, and Parkinson’s disease (33). This study showed that the HT group had higher levels of kynurenine in comparison to the euthyroid group. It was reported that HT leads to the accumulation of kynurenine as a result of a reduction in the activity of kynurenine 3-hydroxylase (34). The depletion in kynurenine levels upon the treatment of HT can be a promising biomarker for monitoring HT therapy. Another altered key metabolite in tryptophan metabolism pathway was tryptamine. Tryptophan is processed by the gut microbiota into tryptamine, also referred to as TrpN. Tryptophan catabolites are absorbed through the intestinal epithelium, enter the bloodstream, and then are eliminated in the urine. Tryptophan can be transformed into tryptamine by both Clostridium and Ruminococcus species, which serves as a precursor for several hormones and neurotransmitters (35). The relationship between autoimmune diseases and modifications in the makeup of gut microbial organisms has been supported by numerous investigations (36–38). Few studies, however, have examined the connection between HT and alterations in gut flora. According to one study, the number of Ruminococcus, Rikenellaceae, Prevotella, Megamonas, and Veillonella strains dropped among HT patients but the number of Bacilli, Lactobacillales, Prevotella, and Megamonas strains increased in HT patients compared to healthy volunteers. Patients with HT also displayed a reduction in intestinal microbial diversity. In this study, the levels of tryptamine have spiked upon the restoration of euthyroid state, as the main source of tryptamine is intestinal microflora; this observation indicates restoration of intestinal microbiota numbers and diversity after the treatment with carbimazole (39).

A balanced redox state is essential for numerous cellular functions, including responses to ROS, signaling, protection of protein thiols, oxidation-reduction reactions, and the elimination of xenobiotics. The entire balance of a cell compartment’s an oxidation/reduction systems determines its redox environment (40). These systems are numerous; therefore the redox environment is the total reduction potential and reduces the capacity of all the redox couples that are present including glutathione/glutathione disulfide and cysteine/cysteine (41). The plasma redox state is mainly determined by the redox status of cysteine/cysteine, which in turn can be utilized to detect oxidative stress in people in a clinical context (42). In the current work, the level of L-cystine was significantly decreased upon treating HT patients with carbimazole. The accumulation of this cysteine dimer is considered a sign of oxidative stress which was reported to accompany HT patients (43); nevertheless, upon restoring the euthyroid state, L-cystine levels dropped; this suggests that the plasma level of L-cystine can be used to monitor the progress of euthyroid state restoration.

## Conclusion

A metabolomic comparison in patients with HT before and after treatment revealed many significantly altered metabolites both up-or downregulated with treatment. A significant decrease in intermediate-chain acylcarnitines and kynurenine and an increase in tryptamine levels were noticed after treatment initiation. This distinctive metabolic pattern has the potential power to help researchers better understand the potential metabolic changes related to HT, enhanced disease detection and maybe thyroid treatment monitoring.

## Data availability statement

The original contributions presented in the study are included in the article/**Supplementary Material**. Further inquiries can be directed to the corresponding authors.

## Ethics statement

The studies involving human participants were reviewed and approved by College of Medicine, King Saud University, Riyadh, Saudi Arabia (registration no. E-10-172. The patients/participants provided their written informed consent to participate in this study.

## Author contributions

AAA, HB, and AM conceived the idea and designed the study. AAA, AM, and AMA were involved in patient recruitment. HB and MM performed the proteomics lab work. HB, AM, MM, and AAA carried out data analysis. AM, HB, MR, MM, and AAA wrote the manuscript. All authors have read and approved the fi-nal manuscript.

## Funding

This project was funded by the National Plan for Science, Technology and Innovation (MAARIFAH), King Abdulaziz City for Science and Technology, Saudi Arabia, (Project No. 08-MED513-02).

## Acknowledgments

We wish to acknowledge Mr. Majed Kenneth and Ms. Amina Fallata for their assistance in the laboratory work.

## Conflict of interest

The authors declare that the research was conducted in the absence of any commercial or financial relationships that could be construed as a potential conflict of interest.

## Publisher’s note

All claims expressed in this article are solely those of the authors and do not necessarily represent those of their affiliated organizations, or those of the publisher, the editors and the reviewers. Any product that may be evaluated in this article, or claim that may be made by its manufacturer, is not guaranteed or endorsed by the publisher.
